# The Chief Operations of Ophthalmic Surgery

**Published:** 1905-03

**Authors:** 


**The Chief Operations of Ophthalmic Surgery.** By HAROLD B. GRIMSDALE, M.B., F.R.C.S. Pp. iv., 144. London: *The Medical Times*, Limited. 1904.

This little book is what it professes to be, namely a practical guide to students of ophthalmology who are intending to undertake the responsibility of operating in this speciality. There is an old saying, perhaps somewhat coarse, that before a surgeon becomes a successful ophthalmic operator he will have probably had a hat-ful of failures, and it is our opinion that careful study of Mr. Grimsdale's book may help to prevent such a calamity. The author does not claim for his work that it is a history of the art. It is essentially a practical guide, and consists of a series of lectures given in connection with his work at the Royal Westminster Ophthalmic Hospital, in which he describes and explains the various operations and enumerates the necessary instruments and arranges the stages of the operations in a way that is concise and clear. To house-surgeons who often get a chance of operating under the guidance of their principals—and we here may go out of our way to recommend them to take every chance of doing so—we recommend the book as likely to be of great use.

The author is in the habit of using for demonstration pigs' heads for operation on the lids and muscles, and pigs' eyes held in a special machine for operation on the globe. This plan may fix in the mind of the student the plan of the procedure, but, as the author points out, the touch of the living eye and the elasticity of the living tissues render the real operation a very different thing.

There are a few grammatical errors, and the work does not claim to be complete, but a future edition, with a preliminary page of contents and more diagrams, and these a little more elaborate, will be welcomed.

